# The Co-Existence of the IL-18+183 A/G and MMP-9 -1562 C/T Polymorphisms Is Associated with Clinical Events in Coronary Artery Disease Patients

**DOI:** 10.1371/journal.pone.0074498

**Published:** 2013-09-05

**Authors:** Trine B. Opstad, Alf Å. Pettersen, Harald Arnesen, Ingebjørg Seljeflot

**Affiliations:** 1 Center for Clinical Heart Research, Department of Cardiology, Oslo University Hospital, Ullevål, Oslo, Norway; 2 Center for Heart Failure Research, Oslo University Hospital, Oslo, Norway; 3 Faculty of Medicine, University of Oslo, Oslo, Norway; University Heart Center, Germany

## Abstract

**Objective:**

Interleukin (IL)-18 has been associated with severity of atherosclerosis and discussed to predict cardiovascular (CV) events. We have previously shown that the IL-18+183 G-allele significantly reduces IL-18 levels. This study was aimed to investigate the prognostic significance of the IL-18+183 A/G polymorphism (rs5744292), single and in coexistence with the matrix metalloproteinase (MMP)-9 -1562 C/T (rs3918242) polymorphism, in patients with stable coronary artery disease (CAD). Serum levels of IL-18, MMP-9 and tissue inhibitor of matrix metalloproteinase (TIMP)-1 were additionally assessed.

**Methods:**

1001 patients with angiographically verified CAD were genotyped and the biomarkers were measured accordingly. After two years follow-up, 10.6% experienced new clinical events; acute myocardial infarction (AMI), stroke, unstable angina pectoris and death.

**Results:**

The IL-18+183 G-allele associated with 35% risk reduction in composite endpoints after adjusting for potential covariates (*p* = 0.044). The IL-18+183 AA/MMP-9 -1562 CT/TT combined genotypes associated with a significant increase in risk of composite endpoints (OR = 1.87; 95% CI = 1.13–3.11, *p* = 0.015, adjusted). Patients with clinical events presented with significantly higher IL-18 levels as compared to patients without (*p* = 0.011, adjusted). The upper tertile of IL-18 levels associated with an increase in risk of AMI as compared to lower tertiles (OR = 2.36; 95% CI = 1.20–4.64, *p* = 0.013, adjusted).

**Conclusion:**

The IL-18+183 A/G polymorphism, single and in combination with MMP-9 genotypes, may influence the risk of clinical events in stable CAD patients.

## Introduction

The pro-inflammatory cytokine interleukin-18 (IL-18) is among the more recently recognized cytokines assumed to be involved in the development of cardiovascular disease (CVD). Increasing evidence from both experimental and clinical studies indicates that IL-18 is a key player in the inflammatory process leading to atherosclerosis [1]–[3]. Furthermore, the augmentation of IL-18 has been associated with different clinical states of CVD; acute coronary syndrome (ACS), type 2 diabetes, the metabolic syndrome, hypertension (HT), worse prognosis of CVD and mortality [4]–[9].

Known stimuli for IL-18 synthesis are hyperglycemia [10], [11], nuclear transcription factor -κB (NFκB) and interferon-gamma (IFNγ) [12], cathecholamines [13], angiotensin II [14] and inflammation in general, whereas IL-18 induces multiple effects downstream by stimulating maturation of T cells and inducing expression of chemokines, adhesion molecules and cytokines, including INFγ and matrix metalloproteinases (MMPs) in different cell types [12], including MMP-9 [15]. Notably, high levels of MMP-9 have been associated with plaque progression, destability and rupture [16]. These various effects exaggerate the inflammatory process, promoting atherosclerosis and increasing the risk of atherothrombosis and cardiovascular (CV) events.

Elevated levels of IL-18 were previously shown to be predictive of coronary events in healthy middle-aged men [17] and in high risk patients [7], CV death in patients with stable and unstable angina pectoris (UAP) [9], and of all-cause mortality in ACS [18]. We and others have previously shown that the IL-18+183 A/G polymorphism, located in the 3′ untranslated region (UTR) of the IL-18 gene mapped to chromosome 11q22 in humans, induces lower levels of circulating IL-18 [19], [20]. The polymorphism is postulated to affect mRNA stability, which may be a molecular basis for the observation. The +183 A-allele was further observed to be more frequent in patients with HT as compared to the variant G-allele [19]. By studying five different IL-18 polymorphisms, which defined six haplotypes, the only haplotype showing a protective effect on CV mortality, included the +183 G allele. The same haplotype was also associated with lower IL-18 levels [20].

IL-18 has been shown to induce MMP-9 expression through the nuclear factor κB (NFκB) pathway [21], thus, combined determination of both markers, also genetically, may be of special interest in relation to risk prediction.

The T-allele of the MMP-9 -1562 C/T promoter polymorphism has been shown in several studies to induce higher MMP-9 levels [22], [23] and is also correlated to clinical severity [24], thus being a potential genetic marker for such combined genetic risk assessment. This polymorphism has been associated with higher promoter activity and increased expression of the gene, due to preferential binding of a transcription repressor to the C-allele [24].The human MMP-9 gene maps to chromosome 20q11-13.

Based on the knowledge that these specific polymorphisms in the IL-18 and MMP-9 genes have the ability to modify the circulating levels of their respective protein, it might be suggested that a combination of these polymorphisms may be of special importance.

We aimed therefore in the present study to explore the predictivity of the IL-18+183 A/G polymorphism, single and in co-existence with the MMP-9 -1562 C/T polymorphism, for clinical events in patients with stable CAD in a follow-up period of 2 years. Circulating levels of IL-18, MMP-9 and TIMP-1 were further assessed in relation to outcome.

The study showed an association of the IL-18+183 A/G polymorphism with new CV events, and the risk increased when both IL-18 and MMP-9 polymorphisms were present. The upper tertile of circulating IL-18 levels was predictive for acute myocardial infarction (AMI).

## Materials and Methods

### Study Population

In the present study 1001 patients with angiographically verified stable CAD were investigated (median age 62 years, 22% women, 97% of Western European descent), all enrolled in the ASCET (ASpirin non-responsiveness and Clopidogrel Endpoint Trial) study [25], [26]. Patients were followed up for a minimum of 2 years and the primary end point included the first event of the composite of nonfatal AMI, unstable angina pectoris (UAP), stroke and all-cause mortality. In patients unable to attend the final visit, the clinical endpoints were recorded on request. No patients were lost to follow-up. Evaluation of end points was performed by an end point committee without access to the laboratory data.

Relatedness in the population was <1%. The ASCET study is registered at; clinicaltrials.gov, identification number: NCT00222261.

### Ethics Statement

The study was approved by The Regional Committee of Medical Research Ethics in South-Eastern Norway. All patients gave their written informed consent to participate.

### Laboratory Methods

In fasting conditions between 8.00–10.00 a.m., blood samples were collected at entrance into the ASCET study. Serum was prepared by centrifugation within 1 hour at 2.500×g for 10 minutes for determination of IL-18, MMP-9 and TIMP-1, and EDTA blood was collected for DNA extraction, all kept frozen at –80°C until analyzed. Routine analyses (lipids, glucose) were measured by use of convential methods. Concentrations of IL-18, MMP-9 and TIMP-1 were determined using the Human IL-18 ELISA kit (Medical Biological Laboratories, Naka-ku Nagoya, Japan), and the Total MMP-9 and TIMP-1 ELISA kits (R&D Systems, Europe, Abingdon, Oxon, UK). The coefficients of variation for the IL-18, MMP-9 and TIMP-1 analysis were 8.1%, 7.3% and 4.4%, respectively. The concentrations of IL-18 MMP-9 and TIMP-1 were analyzed in all patients.

DNA was isolated from whole blood using a MagNA Pure LC DNA Isolation kit (Roche Diagnostics, GmbH, Mannheim, Germany). DNA purity and quantity were tested on the NanoDrop, ND-1000 (Saveen Werner, Sweden). The 3′UTR +183A/G polymorphism (rs5744292) was genotyped with a Custom Design Assay manufactured by Applied Biosystems’ The Custom TaqMan® Assays Service in a 7900 HT Real-Time polymerase chain reaction (PCR) system (Applied Biosystems, Foster City, CA, USA). The MMP-9 -1562 C/T polymorphism (rs3918242) was genotyped by Real-Time PCR and melting curves analysis on the Light Cycler Instrument 1.2 (Roche Diagnostics) using primers 5′GATCACTTGAGTCAGAAGTTCGAAA3′ and 5′TTTGGGGGGTGTAGTATCACTCT3′ and probes synthesized by TIB MOLBIOL (D-12103 Berlin, Germany), and Light Cycler® FastStart DNA Master Hybridization Probes kit (Roche Diagnostics).

The IL-18+183 A/G and MMP-9 -1562 C/T polymorphisms were successfully analyzed in 996 samples. Genotype calling was automatically applied in the assay, with 95% confidence. About 5% of the samples were repeated, with 100% concordance. For the +183 A/G SNP assay, samples with verified AA/AG/GG genotypes were included as positive controls, kindly provided by Dr. Laurence Tirét, Faculté de Médicine, Paris, France.

### Statistics

Students’ *t*- test and Mann-Whitney test, when appropriate, were used for continuous data, and the χ^2^ test for categorical data. For correlation analysis, Spearman’s Rho was applied. The associations of circulating IL-18 and IL-18/MMP-9 genotypes, respectively, with clinical outcome were analyzed by linear and logistic regression models, with adjustment of potential covariates; age, sex, previous MI and stroke, treatment modality (i.e. aspirin or clopidogrel), and the use of nitrates, as appear from Table 1 (*p*<0.2). The Hardy-Weinberg equilibrium (HWE) was tested using the χ^2^ test. All statistical analyses were performed by SPSS 19.0 (SPSS Inc., Chicago, Illinois, USA). A two-tailed probability test of 0.05 or less was considered statistically significant.

**Table 1:** Baseline characteristics according to occurrence of clinical composite endpoints after 2 years.

Baseline characteristics	With endpoints (n = 106)	Without endpoints (n = 895)	*p*
Age (years, mean (range))	63 (41–80)	62 (36–81)	0.499
Men/Women n (%)	83/23 (78/22)	700/195 (78/22)	0.983
Type 2 Diabetes Mellitus n (%)	24 (23)	176 (20)	0.469
Previous myocardial infarction n (%)	57 (54)	380 (43)	0.026
Previous stroke (%)	6 (6)	21 (2.3)	0.047
Hypertension n (%)	63 (59)	493 (55)	0.394
Current smokers n (%)	23 (22)	180 (20)	0.666
BMI (kg/m^2^)	26.8 (24.4, 29.9)*	27.2 (24.9, 29.6)*	0.712
Total cholesterol (mmol/L)	4.5 (1.0)	4.6 (1.0)	0.877
HDL cholesterol (mmol/L)	1.3 (0.4)	1.3 (0.4)	0.898
LDL cholesterol (mmol/L)	2.5 (0.8)	2.5 (0.8)	0.758
Triglycerides (mmol/L)	1.25 (1.01, 1.87)*	1.32 (0.93, 1.84)*	0.866
Fasting glucose (mmol/L)	6.1 (1.7)	6.0 (1.9)	0.914
Medication %			
Statins	98.2	99.1	0.524
β-Blockers	74	76	0.867
Nitrates	27	21	0.145
ACE inhibitors	31	26	0.320
ARB	26	24	0.711
CCB	27	25	0.656
Diuretics	26	22	0.417

Values are mean (SD) or number (proportions) if not otherwise stated. SD: standard deviation, BMI: body mass index, HDL: high density lipoprotein, LDL: low density lipoprotein, ACE: angiotensin converting enzyme, ARB: angiotensin receptor blocker, CCB: calcium channel blocker.

*p*-values are chi-square test for categorical variables and *t*-test or Mann-Whitney test for continuous variables, referring to differences between patients with and without endpoints.

* Median levels (25, 75 percentiles).

## Results

### Characteristics of the Study Population

The total number of primary endpoints recorded was 106; AMI (n = 36), stroke (n = 28), UAP (33) and deaths (n = 9). Baseline characteristics of patients according to occurrence of clinical endpoints are shown in Table 1. Previous myocardial infarction (MI), stroke and nitrates medication were more frequent in patients with new clinical events. Due to low number of deaths, and a heterogeneous group of patients with UAP, these subgroups have not been separately analyzed.

We have previously reported on the influence of the IL-18+183 A/G and MMP-9 -1562 C/T polymorphisms on circulating protein levels [19], [22], showing IL-18 levels to be significantly lower in subjects carrying the G-allele and MMP-9 levels to be significantly higher in subjects carrying the T-allele. Other previously investigated polymorphisms (IL-18 -137 and IL-18 -607 in the IL-18 promoter and the MMP-9 R79Q A/G in exon 6) did not affect circulating protein levels [19], [22].

### Genetic Influence on New Clinical Events

The associations of the IL-18+183 A/G and MMP-9 -1562 C/T polymorphisms with clinical endpoints are shown in Table 2. The IL-18 G-allele associated with 35% risk reduction in composite endpoints and the association persisted after adjustment for covariates (OR = 0.65; 95% confidence interval (CI) = 0.43–0.99, *p* = 0.044). As the reference IL-18 AA genotype (wild-type) seems to be unfavorable, we combined the AA genotype with the MMP-9 -1562 T-allele, known to induce elevated MMP-9 levels, and tested their coexistence to evaluate a common genetic score. The combined IL-18 AA/MMP-9 CT/TT genotypes associated with an increase in risk of composite endpoints (OR = 1.87; 95% CI = 1.13–3.11, *p* = 0.015, adjusted) and ischemic stroke (OR = 2.54; 95% CI = 1.07–6.00, *p* = 0.034, adjusted). The single presence of the -1562 T-allele only tended to associate with composite endpoints. The IL-18 and MMP-9 genotypes were in Hardy Weinberg Equilibrium (*p*>0.4, both) and the observed minor allele frequencies are in line with previous reports.

**Table 2:** Frequencies of the IL-18+183 A/G and MMP-9 -1562 C/T polymorphisms, as related to clinical endpoints.

Endpoints		n	IL-18+183 G-allelefrequency (%)	*p* *	IL-18+183AA/MMP-9−1562 CT/TT n (%)	*p* *	MMP-9 −1562 T-allelefrequency (%)	*p* *
Composite	Yes	106	0.22 (36.7)	0.044#	23 (22)	0.015†	0.16 (31.1)	0.075
	No	890	0.26 (46.5)		113 (13)		0.13 (23.3)	
AMI	Yes	36	0.21 (33.3)	0.144	6 (17)	>0.2	0.14 (27.8)	>0.2
	No	960	0.26 (45.9)		130 (14)		0.13 (24.0)	
Stroke	Yes	28	0.23 (32.2)	0.121	8 (29)	0.034‡	0.18 (35.7)	0.151
	No	968	0.26 (45.9)		128 (13)		0.13 (23.7)	

*p*-values refer to difference in frequencies of the IL-18/MMP-9 polymorphisms alone and in combination, as related to clinical endpoints.

* Adjusted for age, gender, previous MI, stroke, treatment modality, and use of nitrates.

# OR 0.65 (95% CI 0.43, 0.99).

† OR 1.87 (95% CI 1.13, 3.11).

‡ OR 2.54 (95% CI 1.07, 6.00).

### Serum Concentrations and Future Clinical Events

**Figure 1:**
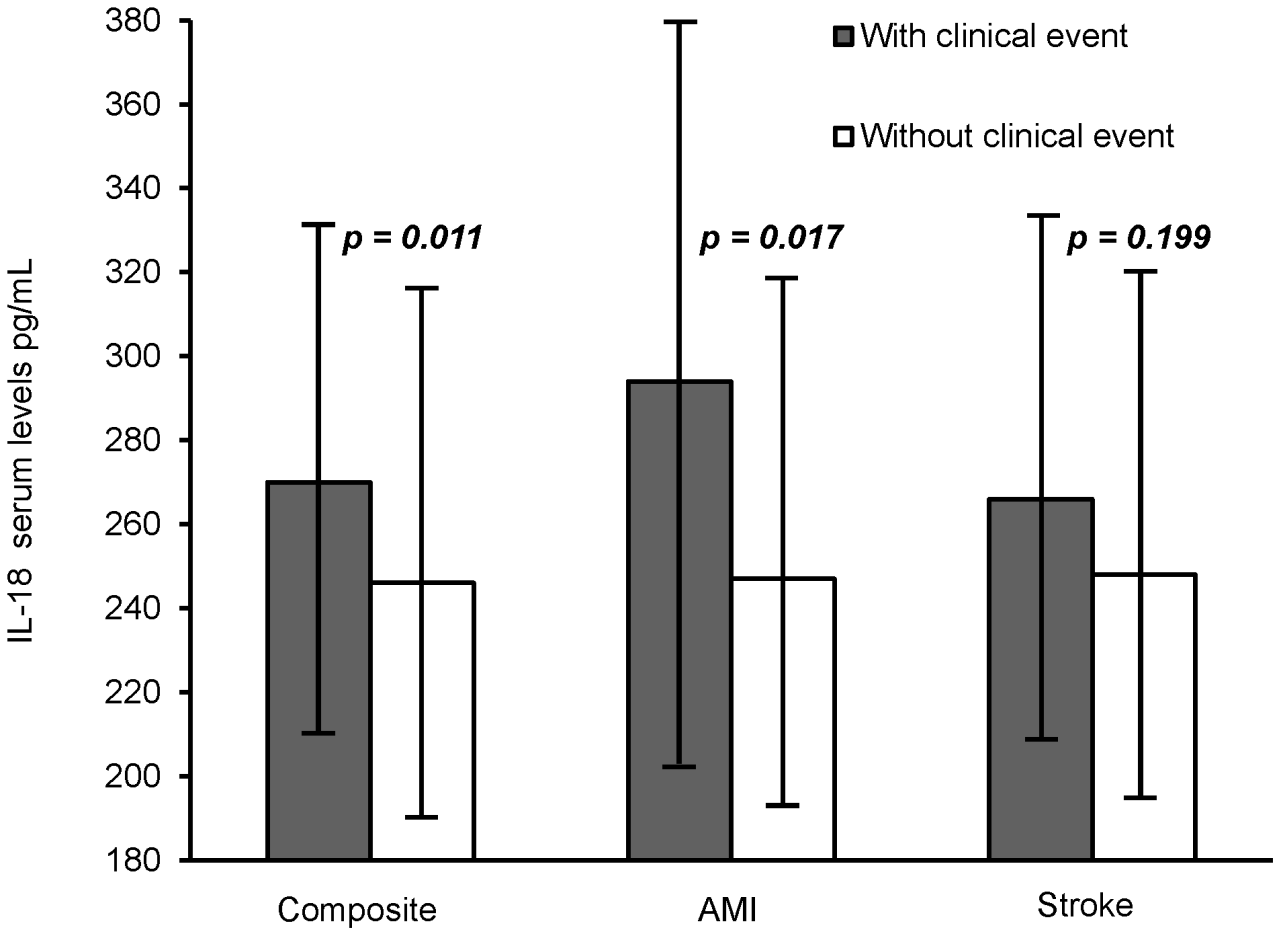
Median levels of IL-18 (25, 75 percentiles) as related to clinical endpoints. p-values are adjusted for age, gender, previous myocardial infarction (MI), stroke, treatment modality, and use of nitrates.

**Figure 2:**
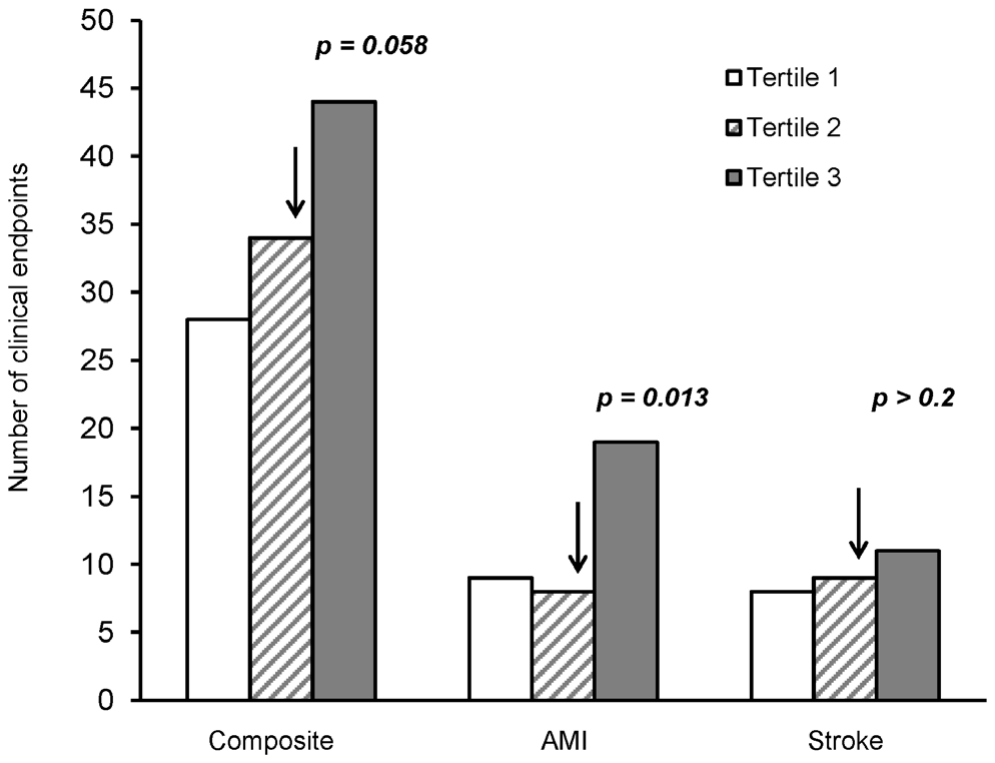
Tertiles of IL-18 as related to clinical endpoints. 33 percentiles = 212.5 pg/mL, 66 percentiles = 293.1 pg/mL. p-values refer to the comparison of 2 groups dichotomized between second and third tertile. p-values are adjusted for age, gender, previous myocardial infarction (MI) and stroke, treatment modality and use of nitrates.

As shown in Figure 1, serum levels of IL-18 were significantly higher in patients with clinical endpoints during follow-up as compared to patients without (270 versus 246 pg/mL, *p* = 0.011, adjusted) and especially higher in patients suffering AMI (294 versus 247 pg/mL, *p* = 0.017, adjusted). Levels of IL-18 were not significantly elevated in patients with ischemic stroke. When dividing IL-18 levels into tertiles, a significant trend for increasing number of AMI through tertiles was observed (*p* for trend = 0.038) (Figure 2). The upper tertile of IL-18 was strongly associated with incidence of AMI as compared to the two lowest tertiles, OR = 2.36, 95% CI = 1.20–4.64, *p* = 0.013, adjusted. When additionally adjusting for the IL-18+183 polymorphism, the association persisted (OR = 2.30; 95% CI = 1.16–4.54, *p* = 0.017). The upper tertile associated similarly with the incidence of composite endpoints, OR = 1.50, 95% CI = 0.99–2.27, however only borderline significant (*p* = 0.058), and was not associated with stroke.

Serum levels of MMP-9 and TIMP-1 were not related to future clinical events in either of the endpoint groups (data not shown). Notably, IL-18 levels above median associated with significantly higher MMP-9 levels as compared to patients with IL-18 levels below median (MMP-9∶243 versus 230 pg/mL, *p* = 0.027, adjusted). The two biomarkers were only weakly correlated (r = 0.077, *p* = 0.019).

The additive effect of having the combined IL-18 AA and MMP-9 CT/TT genotypes on circulating protein levels was not statistically significant, although numerically higher levels were observed of both markers (IL-18∶258 versus 246 pg/mL, adjusted p-value = 0.105, and for MMP-9∶257 versus 234 pg/mL, adjusted p-value = 0.057), when compared to the combined IL-18 AG/GG/MMP-9 CC genotypes.

## Discussion

In this prospective study of patients with known stable CAD, we have observed an association between the IL-18+183 A/G polymorphism and an increased risk of future clinical events in a time-frame of minimum two years. The importance of circulating IL-18 levels in predicting new events is additionally verified, especially with regard to future AMI. We further observed an additional risk of composite endpoints when combining IL-18/MMP-9 genotypes. The observed associations were unaffected by adjustment for clinical and therapeutic covariates.

In a population comparable to ours, the IL-18+183 A/G polymorphism, in haplotypes with other linked IL-18 polymorphisms, has previously been shown associated with CV mortality during 4 years of follow-up [20]. Out of 6, the only haplotype that was related to reduced mortality risk included the +183 G-allele [20]. In the present study the +183 G-allele associated with 35% risk reduction of composite endpoints, and 6 out of 9 deaths were homozygous of the +183 A-allele (data not shown). We have previously reported on the lack of an association between two promoter IL-18 polymorphisms and IL-18 serum concentrations [19]. The two variants, −137 G/C (rs187238) and −607 C/A (rs1946518) were also not associated with clinical endpoints in the present study. The −137 G/C polymorphism was previously shown to associate with the occurrence of sudden cardiac death among Western European descent males [27].

Little is known about the combined genetic influence of IL-18 and MMP-9, which may provide additional information on CV risk and prediction, as previously indicated [23]. IL-18 has been shown to release MMP-9 from peripheral blood mononuclear cells [28] and the T helper cell 1 polarization driven by IL-18 may also affect MMP-9 expression [29]. Both IL-18 and MMP-9 are considered as important mediators during the development of CVD, and the combined determination of IL-18 and MMP-9, also genetically, may identify patients at very high risk. We have demonstrated for the first time that patients genetically predisposed for elevated circulating levels of both biomarkers, possessed additionally higher risk of composite clinical endpoints.

The risk prediction of circulating IL-18 levels has previously been demonstrated regarding CV mortality [9], heart failure, MI and deaths in patients diagnosed with ACS [18], and in healthy men suffering coronary events [17]. We have in the present study verified the prognostic value of IL-18 levels for clinical events in stable CAD patients. Elevated IL-18 levels seemed to especially predict AMI, independent of the IL-18+183 A/G polymorphism. Levels of IL-18 were not associated with stroke, probably due to the lower number.

Elevated circulating levels and expression of MMP-9 have been associated with the development and progression of atherosclerosis, and the importance of the MMP-9 gene, especially the functional promoter −1562 C/T polymorphism, is documented in both experimental and clinical studies [24], [30], [31]. Limited and conflicting data exist on the prognostic value of the polymorphism as related to CV outcome [23], [32]. In the present study the −1562 T allele was only weakly associated with new clinical events. The previous investigated MMP-9 exon 6 R279Q A/G (rs17576) polymorphism [22] was not associated with clinical endpoints. We observed no association of MMP-9 circulating levels with clinical outcome, which is in contrast to observations made by Blankenberg et al, observing higher MMP-9 levels in relation to CV death [23]. This discrepancy may be due to differences in study cohorts. Serum concentrations of IL-18 and MMP-9 were only weakly correlated, although statistically significant, suggesting independent functional roles of the two biomarkers with regard to CVD and clinical outcome. The higher MMP-9 levels in patients with IL-18 levels above median and not below, may indicate the need for a certain threshold of IL-18 levels to affect MMP-9 expression.

Our study is limited by the relative short follow-up time and the low number of clinical events. This investigation is a sub-study of the ASCET trial and power calculation was performed on the hypothesis in the main study [25]. A post-hoc power calculation for our purpose could be implemented, however, as the present investigation is restricted to exact number of patients enrolled in the main study, an adequate sample size is unrealizable. The low number of clinical events also contributes to an eventual underestimation. As the present study was performed in Western European descents, the generalization to other ethnical groups should be avoided.

In conclusion, the IL-18+183 A/G polymorphism may be a novel prognostic marker for future clinical events in CAD patients. The additional risk observed for the combined genetic influence of both IL-18/MMP-9 loci suggests the superiority of screening more than one marker to identify patients at high risk. This may provide extra information for CV risk stratification and prediction, although results are considered hypothesis generating and should be replicated in further studies. Finally, the prognostic value of serum IL-18 concentration has previously been documented, and is hereby confirmed in a large cohort of stable CAD patients.
